# Systematic review of intervertebral disc repair: a bibliometric analysis of the 100 most-cited articles

**DOI:** 10.1186/s13018-021-02303-x

**Published:** 2021-03-22

**Authors:** Gang Xu, Xianglong Meng, Juan Guan, Yaozhong Xing, Zihe Feng, Yong Hai

**Affiliations:** 1 Beijing Chao-yang Hospital, Capital Medical University, 8 Gongren Tiyuchang Nanlu, Chaoyang District, Beijing, 100020 China; 2 International Research Center for Advanced Structural and Biomaterials, School of Materials Science and Engineering, Beihang University, Beijing, 100191 China

## Abstract

**Study design:**

A bibliometric review of the literature.

**Objective:**

To identify the most frequently cited articles relating to the repair of intervertebral disc (IVD) and to summarize the key points and findings of these highly cited works, to quantify their impact on the developments of the disc disease treatment.

**Summary of background data:**

IVD repair is an ever-growing and multi-disciplinary innovating treatment method for disc diseases. There are numerous literatures and related studies about it, promoting the development of the field. A comprehensive review and analysis of the most influential articles can help clarify the most effective strategy of IVD repair, and discover the promising directions for future research.

**Methods:**

The Thomson Reuters Web of Knowledge was searched for citations of all literatures relevant to IVD repair. The number of citations, key points, categories, authorships, years, journals, countries, and institutions of publications were analyzed.

**Results:**

The most highly cited articles in IVD Repair were published over 30 years, between 1991 and 2017. Most works (No. 41) were published between 2005 and 2009. The most-cited article was Sakai’s 2003 article which described the possibility of combining MSC and gel to repair IVD. The three most popular categories involved were Orthopedics [44], Clinical Neurology [34], Engineering, and Biomedical [24]. The three most common topics were regenerative medicine and the progenitor cells [33], biomaterials and cellular scaffolds [29], application of growth factors [25]. Author Masuda and the partners have 4 articles in the top 100 list. The Rush University has 12 articles in the top 100 list.

**Conclusion:**

This report identifies the top 100 articles in IVD repair and acknowledges those individuals who have contributed the most to the study of the IVD repair and the body of knowledge used to the repair strategy making. It allows insight into the trends of this innovative and interdisciplinary subspecialty of spine surgery.

## Introduction

The pain and dysfunction of lumber and lower extremities caused by degenerative disc disease (DDD) are the focus of spinal surgery [1]. However, when conservative treatment fails, aggressive treatment is surgical intervention, such as discectomy, disc replacement and spinal fusion, These treatments can only temporarily relieve pain, cannot restore the normal structure and biomechanical function of the IVD, resulting in the loss of normal function of the corresponding segment, and with a higher postoperative recurrence rate. Driven by biomaterials and tissue engineering, it is possible to construct intervertebral discs in vitro and repair in vivo. The WHO has nominated the development of mesenchymal stem cells and cell scaffold to promote IVD repair as primary research objectives [2]. Recent years, basic sciences and clinical research remain paramount in the understanding and advancement of IVD repair, in which many related literatures have been reported. The disciplines involved, research directions, objectives and main ideas, methods about these studies are different from each other. It is difficult to determine the best engineering strategy that may advance IVD repair. For innovative topics, it is particularly important to find research trends and directions during the important articles.

Bibliometric analysis is a method used to study published articles citation history, which can be used to overcome the evaluation mentioned above [3]. The number of citations received by an article can be used to quantify the influence the work has had in a particular area of investigation [4]. The most highly cited publications represent the highest impact work in a given field and serve as the basic of a new category [5]. The research directions of these articles can show the trends in this research area [6]. The greater the number of citations an author has, the more influence they have in their particular area of expertise [7]. As the impact factor (IF) is considered to be a measure of journal quality and rank, the IF of the journal can be analyzed by citation analysis to evaluate the importance of the journal [8].

In spinal surgery, recent publications identified the top 100 articles about spinal deformity surgery [9], imaging of the spine [10], cervical spine surgery [11], lumbar spine surgery [12], and in spinal journals etc. The goal of this article is to identify the most-cited literatures about IVD repair and to analyze the top 100 of them. They were analyzed around the following parameters: frequency of citation, categories, chronological, geographical, institutional, and journals, as well as the keywords and subject terms involved and their frequency of occurrence. In order to discover the authoritative trends and directions in the research of IVD repair.

## Materials and methods

The Thomson Reuters Web of Knowledge, a search platform that provides bibliographic database services, was used to search for citations of all articles from 1980 to 2019 relevant to the IVD repair. The decision on which journals to search was made with the use of Thomson Reuters Journal Citation Report database, which ranks journals according to impact factor.

The search limits and sorting options in the Thomson Reuters Web of Knowledge were used to rank all articles from each journal according to the number of citations. The results were then carefully reviewed and only those relevant to IVD repair were selected. The 100 articles that matched the search criteria were then further analyzed. Specifically, the title, first author, journal, year of publication, number of citations, origin nations and institutions were recorded.

## Result

**Fig. 1:**
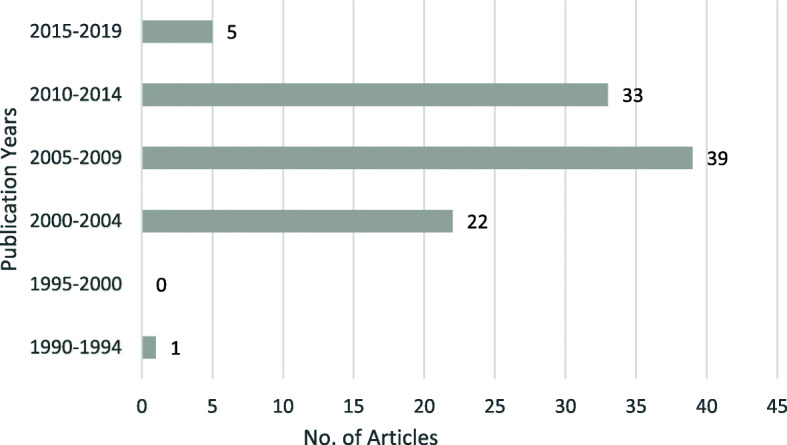
The 100 most-cited articles related to IVD repair distributed over 5-year intervals according to date of publication

**Fig. 2:**
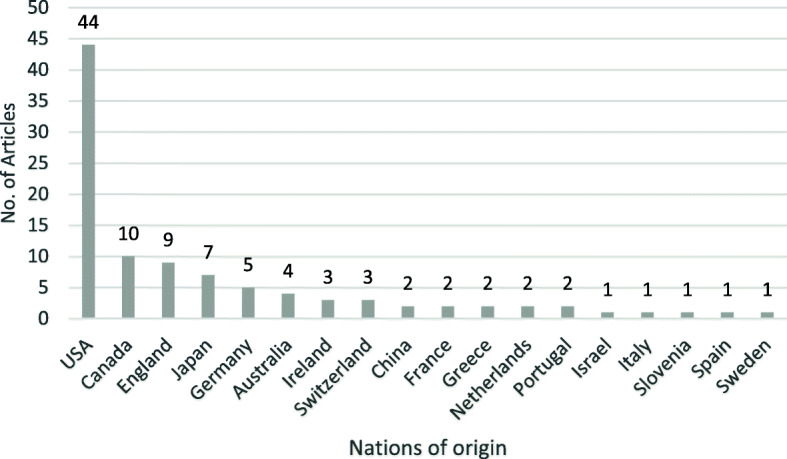
The nations of origin (according to address provided by the first author) for the 100 most-cited articles

A total of 1183 articles matched the search criteria. Of those, 50 were cited more than 100 times. Table 1 demonstrates the top 100 most-cited articles regarding IVD repair. The articles are published between 2000 and 2017 except one is published at 1991, which was written by Thompson and was cited 316 times. The mean number of citations of the selected 100 articles was 112.32. The most highly cited article was “Transplantation of mesenchymal stem cells embedded in Atelocollagen((R)) gel to the intervertebral disc: a potential therapeutic model for disc” published in the Biomaterials by Sakai et al, with 332 citations on Web Of Science (WOS).Between 2005 and 2009, producing the largest number of highly cited articles published (41%) (Fig. 1). The top 100 articles were published in 48 journals, with the top 3 journals publishing 44% of the articles (Table 2). The top journal was Spine, with 23 articles, followed by the Biomaterials, with 9 articles, and the European Spine Journal, with 9 articles. The 3 most popular categories published were Orthopedics (No. is 44), Clinical Neurology (No. is 34), Engineering, Biomedical (No. is 24) (Table 3). A total of 81 first authors contributed to the top 100 articles. Only 2 were credited with 3 or more publications and only 1, Masuda, had 4 publications in the top 100 (Table 4). The top articles originated from 18 different countries, with the USA (44%) being the most prolific (Fig. 2). There were 59 institutions responsible for the top-cited articles with Rush University in Chicago, the USA, contributed 12 publications of the top 100 (Table 5).

**Table 1:** The top 100 papers in IVD repair

Rank	Year	First author	Article title	Key-point	Cited times
1	2003	Sakai, D	Transplantation of mesenchymal stem cells embedded in Atelocollagen((R)) gel to the intervertebral disc: a potential therapeutic model for disc degeneration	Mesenchymal Stem Cell (Msc)	332
2	1991	Thompson, JP	Stimulation of mature canine intervertebral disc by growth factors	Growth Factor	316
3	2006	Sakai, D	Regenerative effects of transplanting mesenchymal stem cells embedded in atelocollagen to the degenerated intervertebral disc	MSC	299
4	2004	Risbud, MV	Differentiation of mesenchymal stem cells towards a nucleus pulposus-like phenotype in vitro: implications for cell-based transplantation therapy	MSC	283
5	2011	Orozco, L	Intervertebral Disc Repair by Autologous Mesenchymal Bone Marrow Cells: A Pilot Study	MSC	238
6	2007	Risbud, MV	Evidence for skeletal progenitor cells in the degenerate human intervertebral disc	Endogenous Progenitors	208
7	2008	Richardson, SM	Human mesenchymal stem cell differentiation to NP-like cells in chitosan-glycerophosphate hydrogels	Chitosan-Glycerophosphate and Msc	202
8	2003	Alini, M	The potential and limitations of a cell-seeded collagen/hyaluronan scaffold to engineer an intervertebral disc-like matrix	Scaffold and Cell	201
9	2004	Mizuno, H	Tissue-engineered composites of anulus fibrosus and nucleus pulposus for intervertebral disc replacement	Novel Materials	199
10	2004	Walsh, AJL	In vivo growth factor treatment of degenerated intervertebral discs	Grow Factor	197
11	2003	Hunter, CJ	The notochordal cell in the nucleus pulposus: A review in the context of tissue engineering	Notochordal Cell	196
12	2003	Ganey, T	Disc chondrocyte transplantation in a canine model: A treatment for degenerated or damaged intervertebral disc	Autologous Chondrocyte	194
13	2007	Meisel, HJ	Clinical experience in cell-based therapeutics: Disc chondrocyte transplantation - A treatment for degenerated or damaged intervertebral disc	Autologous Cultured Disc-Derived Chondrocytes (ADCT)	181
14	2006	Sontjens, SHM	Biodendrimer-based hydrogel scaffolds for cartilage tissue repair	Hydrogel Scaffold	169
15	2011	Smith, LJ	Degeneration and regeneration of the intervertebral disc: lessons from development	Embryonic Morphogenesis	167
16	2004	Masuda, K	Growth factors and treatment of intervertebral disc degeneration	Growth Factor	166
17	2002	Kroeber, M	New in vivo animal model to create intervertebral disc degeneration and to investigate the effects of therapeutic strategies to stimulate disc regeneration	Factor and Signal	155
18	2002	Cs-Szabo, G	Changes in mRNA and protein levels of proteoglycans of the anulus fibrosus and nucleus pulposus during intervertebral disc degeneration	Proteoglycans	151
19	2010	Yoshikawa, T	Disc Regeneration Therapy Using Marrow Mesenchymal Cell Transplantation A Report of Two Case Studies	MSC	149
20	2010	Richardson, SM	Mesenchymal Stem Cells in Regenerative Medicine: Opportunities and Challenges for Articular Cartilage and Intervertebral Disc Tissue Engineering	MSC	147
21	2006	Chujo, T	Effects of growth differentiation factor-5 on the intervertebral disc - In vitro bovine study and in vivo rabbit disc degeneration model study	Growth Factor : rhGDF-5	146
22	2002	Roughley, PJ	The role of proteoglycans in aging, degeneration and repair of the intervertebral disc	Proteoglycan	142
23	2015	Rosenzweig, DH	3D-Printed ABS and PLA Scaffolds for Cartilage and Nucleus Pulposus Tissue Regeneration	Hydrogel Scaffold and MSC	135
24	2008	Nesti, LJ	Intervertebral disc tissue engineering using a novel hyaluronic acid-nanofibrous scaffold (HANFS) amalgam	Scaffold	135
25	2009	Bron, JL	Repair, regenerative and supportive therapies of the annulus fibrosus: achievements and challenges	MSC	134
26	2013	Iatridis, JC	Role of biomechanics in intervertebral disc degeneration and regenerative therapies: what needs repairing in the disc and what are promising biomaterials for its repair?	Biomechanics	134
27	2009	Ganey, T	Intervertebral Disc Repair Using Adipose Tissue-Derived Stem and Regenerative Cells Experiments in a Canine Model	Adipose Tissue-Derived Stem and Regenerative Cells (ADRC)	133
28	2007	Chubinskaya, S	OP-1/BMP-7 in cartilage repair	BMP-7 and Osteogenic protein-1(OP-1)	133
29	2011	Collin, EC	An injectable vehicle for nucleus pulposus cell-based therapy	Hydrogel and ADRC	129
30	2008	Masuda, K	Biological repair of the degenerated intervertebral disc by the injection of growth factors	Review: Annulus Ribrous Repair	128
31	2016	Richardson, SM	Mesenchymal stem cells in regenerative medicine: Focus on articular cartilage and intervertebral disc regeneration	Growth Factor	128
32	2005	Zhang, YG	Bone mesenchymal stem cells transplanted into rabbit intervertebral discs can increase proteoglycans	MSC	127
33	2008	Hohaus, C	Cell transplantation in lumbar spine disc degeneration disease	ADCT	126
34	2013	Guterl, CC	CHALLENGES AND STRATEGIES IN THE REPAIR OF RUPTURED ANNULUS FIBROSUS	Cells, Scaffold and Signal	126
35	2007	O'Halloran, DM	Tissue-engineering approach to regenerating the intervertebral disc	Cells, Scaffold and Signal	119
36	2012	Pattappa, G	Diversity of intervertebral disc cells: phenotype and function	IVD Cell Phenotype	117
37	2002	Takegami, K	Osteogenic protein-1 enhances matrix replenishment by intervertebral disc cells previously exposed to interleukin-1	Interleukin-1(IL1α)+OP1	115
38	2008	Ellman, MB	Biological impact of the fibroblast growth factor family on articular cartilage and intervertebral disc homeostasis	Cell Phenotype and MSC	114
39	2010	Strassburg, S	Co-culture induces mesenchymal stem cell differentiation and modulation of the degenerate human nucleus pulposus cell phenotype	Growth Factor	114
40	2008	Wan, YQ	Biphasic scaffold for annulus fibrosus tissue regeneration	Scaffold	113
41	2001	Johnson, WEB	Cell cluster formation in degenerate lumbar intervertebral discs is associated with increased disc cell proliferation	IVD Cell Phenotype	111
42	2001	Baer, AE	Collagen gene expression and mechanical properties of intervertebral disc cell-alginate cultures	PNCA / KI67	111
43	2004	Gorensek, M	Nucleus pulposus repair with cultured autologous elastic cartilage derived chondrocytes	ADCT	105
44	2003	An, HS	Biological repair of intervertebral disc	MSC	104
45	2007	Paesold, G	Biological treatment strategies for disc degeneration: potentials and shortcomings	Gene Therapy and Tissue Engineering	103
46	2008	Korecki, CL	Needle puncture injury affects intervertebral disc mechanics and biology in an organ culture model	Biomechanic	102
47	2005	Perie, D	Confined compression experiments on bovine nucleus pulposus and annulus fibrosus: sensitivity of the experiment in the determination of compressive modulus and hydraulic permeability	Biomechanic	101
48	2009	McGirt, MJ	A Prospective Cohort Study of Close Interval Computed Tomography and Magnetic Resonance Imaging After Primary Lumbar Discectomy Factors Associated With Recurrent Disc Herniation and Disc Height Loss	Lumbar Discectomy	100
49	2003	Sato, M	An atelocollagen honeycomb-shaped scaffold with a membrane seal (ACHMS-scaffold) for the culture of annulus fibrosus cells from an intervertebral disc	Scaffold	99
50	2005	Mwale, F	Biological evaluation of chitosan salts cross-linked to genipin as a cell scaffold for disk tissue engineering	Scaffold	98
51	2010	Shen, BJ	The Role of BMP-7 in Chondrogenic and Osteogenic Differentiation of Human Bone Marrow Multipotent Mesenchymal Stromal Cells In Vitro	BMP-7 and MSC	97
52	2008	Wuertz, K	Behavior of mesenchymal stem cells in the chemical microenvironment of the intervertebral disc	Inflammatory Response	96
53	2015	Molinos, M	Inflammation in intervertebral disc degeneration and regeneration	MSC	96
54	2014	Huang, YC	OPINION Intervertebral disc regeneration: do nutrients lead the way?	Disc Nutrients Supply	96
55	2011	Grunhagen, T	Intervertebral Disk Nutrition: A Review of Factors Influencing Concentrations of Nutrients and Metabolites	Disc Nutrients Supply	95
56	2006	Iatridis, JC	Effects of mechanical loading on intervertebral disc metabolism in vivo	Mechanical Loading and Disc-cell Metabolism	94
57	2002	Alini, M	A biological approach to treating disc degeneration: not for today, but maybe for tomorrow	Biomatrix	93
58	2005	Kroeber, M	Effects of controlled dynamic disc distraction on degenerated intervertebral discs - An in vivo study on the rabbit lumbar spine model	In Vivo Model	93
59	2006	Akeda, K	Platelet-rich plasma (PRP) stimulates the extracellular matrix metabolism of porcine nucleus pulposus and anulus fibrosus cells cultured in alginate beads	Platelet-rich Plasma(PRP) and Growth Factor	93
60	2009	Shen, BJ	BMP-2 Enhances TGF-beta 3-Mediated Chondrogenic Differentiation of Human Bone Marrow Multipotent Mesenchymal Stromal Cells in Alginate Bead Culture	Disc Distraction	92
61	2007	Chang, G	Porous silk scaffolds can be used for tissue engineering annulus fibrosus	Scaffold	92
62	2010	Korecki, CL	Notochordal cell conditioned medium stimulates mesenchymal stem cell differentiation toward a young nucleus pulposus phenotype	NC and MSC for NP Cell Phenotype	91
63	2006	Boyd, LM	Injectable biomaterials and vertebral endplate treatment for repair and regeneration of the intervertebral disc	Scaffold	89
64	2007	Revell, PA	Tissue engineered intervertebral disc repair in the pig using injectable polymers	Scaffold and MSC	89
65	2010	Calderon, L	TYPE II COLLAGEN-HYALURONAN HYDROGEL - A STEP TOWARDS A SCAFFOLD FOR INTERVERTEBRAL DISC TISSUE ENGINEERING	Scaffold and MSC	89
66	2015	Dimozi, A	OXIDATIVE STRESS INHIBITS THE PROLIFERATION, INDUCES PREMATURE SENESCENCE AND PROMOTES A CATABOLIC PHENOTYPE IN HUMAN NUCLEUS PULPOSUS INTERVERTEBRAL DISC CELLS	Oxidative stress	88
67	2010	Kallewaard, JW	Discogenic Low Back Pain	Low Back Pain	87
68	2005	Takegami, K	Osteogenic protein-1 is most effective in stimulating nucleus pulposus and annulus fibrosus cells to repair their matrix after chondroitinase ABC-induced in vitro chemonucleolysis	OP-1	86
69	2011	Schek, RM	GENIPIN-CROSSLINKED FIBRIN HYDROGELS AS A POTENTIAL ADHESIVE TO AUGMENT INTERVERTEBRAL DISC ANNULUS REPAIR	Novel Materials	85
70	2010	Otsuki, S	Extracellular sulfatases support cartilage homeostasis by regulating BMP and FGF signaling pathways	Growth factor and Signal	83
71	2005	Magne, D	Mesenchymal stem cell therapy to rebuild cartilage	MSC	83
72	2007	Imai, Y	Restoration of disc height loss by recombinant human osteogenic protein-1 injection into intervertebral discs undergoing degeneration induced by an intradiscal injection of chondroitinase ABC	NC	79
73	2003	Hunter, CJ	The three-dimensional architecture of the notochordal nucleus pulposus: novel observations on cell structures in the canine intervertebral disc	OP-1	79
74	2013	Ellman, MB	Fibroblast growth factor control of cartilage homeostasis	ADCT and MSC	78
75	2011	Purmessur, D	Notochordal conditioned media from tissue increases proteoglycan accumulation and promotes a healthy nucleus pulposus phenotype in human mesenchymal stem cells	Growth Factor	78
76	2011	Acosta, FL	Porcine Intervertebral Disc Repair Using Allogeneic Juvenile Articular Chondrocytes or Mesenchymal Stem Cells	MSC	78
77	2006	Masuda, K	Prevention of disc degeneration with growth factors	Growth Factor	75
78	2013	Frith, JE	An injectable hydrogel incorporating mesenchymal precursor cells and pentosan polysulphate for intervertebral disc regeneration	Directly Repair	72
79	2000	Ahlgren, BD	Effect of anular repair on the healing strength of the intervertebral disc - A sheep model	Hydrogel Scaffold and MPC (mesenchymal precursor cell)	72
80	2006	Wilda, H	In vitro studies of annulus fibrosus disc cell attachment, differentiation and matrix production on PDLLA/45S5 Bioglass (R) composite films	Aggrecan	69
81	2014	Sivan, SS	Structure, function, aging and turnover of aggrecan in the intervertebral disc	Composite Film	69
82	2012	Whatley, BR	Intervertebral disc (IVD): Structure, degeneration, repair and regeneration	IVD Regeneration	65
83	2006	Iwashina, T	Low-intensity pulsed ultrasound stimulates cell proliferation and proteoglycan production in rabbit intervertebral disc cells cultured in alginate	Proteoglycan	63
84	2015	Tsaryk, R	Collagen-low molecular weight hyaluronic acid semi-interpenetrating network loaded with gelatin microspheres for cell and growth factor delivery for nucleus pulposus regeneration	Hydrogel	62
85	2006	Masuoka, K	Tissue engineering of articular cartilage with autologous cultured adipose tissue-derived stromal cells using atelocollagen honeycomb-shaped scaffold with a membrane sealing in rabbits	Scaffold and ATSC	61
86	2010	Chang, G	Enhancing annulus fibrosus tissue formation in porous silk scaffolds	Scaffold	60
87	2013	Hudson, KD	Recent advances in biological therapies for disc degeneration: tissue engineering of the annulus fibrosus, nucleus pulposus and whole intenrertebral discs	Scaffold	60
88	2004	Masuda, K	Growth factors and the intervertebral disc	Growth Factor	58
89	2013	Francisco, AT	Injectable laminin-functionalized hydrogel for nucleus pulposus regeneration	Hydrogel	58
90	2012	Chan, SCW	Intervertebral disc regeneration or repair with biomaterials and stem cell therapy - feasible or fiction?	Hydrogel and MSC	58
91	2008	Abbushi, A	Regeneration of intervertebral disc tissue by resorbable cell-free polyglycolic acid-based implants in a rabbit model of disc degeneration	PGA(polyglycolic acid) Implant	58
92	2001	Pattison, ST	Regulation of gelatinase-A (MMP-2) production by ovine intervertebral disc nucleus pulposus cells grown in alginate bead culture by transforming growth factor-beta(1) and insulin like growth factor-I	Growth Factor: MMP and TGF-β1	57
93	2011	Mwale, F	The efficacy of Link N as a mediator of repair in a rabbit model of intervertebral disc degeneration	Synthetic Peptides(link N)	56
94	2009	Mavrogonatou, E	High osmolality activates the G1 and G2 cell cycle checkpoints and affects the DNA integrity of nucleus pulposus intervertebral disc cells triggering an enhanced DNA repair response	DNA repair	55
95	2012	Milani, AH	Injectable Doubly Cross-Linked Microgels for Improving the Mechanical Properties of Degenerated Intervertebral Discs	Scaffold	54
96	2013	Salgado, AJ	Tissue Engineering and Regenerative Medicine: Past, Present, and Future	Scaffold and ADCT	54
97	2005	Masuoka, K	Tissue engineering of articular cartilage using an allograft of cultured chondrocytes in a membrane-sealed atelocollagen honeycomb-shaped scaffold (ACHMS scaffold)	TERM	54
98	2012	Vadala, G	Bioactive electrospun scaffold for annulus fibrosus repair and regeneration	Gene and Protein Expression	53
99	2017	Dowdell, J	Intervertebral Disk Degeneration and Repair	PRP and Growth Factor	53
100	2013	Brisby, H	The Presence of Local Mesenchymal Progenitor Cells in Human Degenerated Intervertebral Discs and Possibilities to Influence These In Vitro: A Descriptive Study in Humans	Scaffold and MSC	53

**Table 2:** Top journals of publication

Journal	No. of articles	Impact factor*
Spine	23	2.903
Biomaterials	9	10.273
European Spine Journal	9	2.513
Tissue Engineering	6	3.508
European Cells & Materials	4	3.682
Journal of Biomedical Materials Research Part A	4	3.221
Arthritis Research & Therapy	3	4.1418
*As of 2019.

**Table 3:** Most popular categories ranked by numbers of articles

Category	No. of articles
Orthopedics	44
Clinical Neurology	34
Engineering, Biomedical	24
Materials Science, Biomaterials	23
Cell & Tissue Engineering	14
Cell Biology	14
Biochemistry & Molecular Biology	13
Surgery	5
Anatomy & Morphology	5
Immunology	4
Rheumatology	4
Biochemical Research Methods	3
Medicine, Research & Experimental	3
Genetics & Heredity	3

**Table 4:** Top authors of publication

Author	No. of articles
Masuda, K	4
Richardson, SM	3
Risbud, MV	2
Chang, G	2
Korecki, CL	2
Ganey, T	2
Iatridis, JC	2
Mwale, F	2
Alini, M	2
Ellman, MB	2
Hunter, CJ	2
Kroeber, M	2
Masuoka, K	2
Sakai, D	2
Shen, BJ	2
Takegami, K	2

**Table 5:** Top institutions of origin of articles

Institution	No. of articles
Rush University	12
McGill University	5
Icahn School of Medicine at Mount Sinai	4
University of California	4
University of Manchester	4
University of Vermont	4
Duke University	3
National Defense medical college	3
National University of Ireland	3
Tokai University	3
BG Hospital Bergmannstrost Halle	2
Cornell University	2
Medical Center Atlanta	2
National center for scientific research “Demokritos”	2
Thomas Jefferson University	2
University of Calgary	2

## Discussion

Bibliometrics is a form of statistical analysis used to quantify the frequency of citations within published academic literature [13]. Although not the sole marker of an articles scientific quality, the number of citations amassed by a paper can be used as a surrogate marker of the impact made within its field.

This article identifies the authors and topics that have made the most impact on the practice of IVD repair over the course of the past 30 years. By identifying these classic works, insight is gained into the history, development, and current trends in IVD repair. The findings of this study identified the articles responsible for the most important developments in this field. Through bibliometrics, we screened the most-cited articles, and we were able to observe that these articles are most often centered around topics that are closely related to tissue engineering regenerative medicine, firstly, the induction of differentiation of stem cells that can achieve maximum restoration of the in vivo structure, so the application of bone marrow mesenchymal stem cells became the most involved topic. Secondly, since the intervertebral disc provides certain biomechanical functions and is a passive load-bearing structure that requires a certain modulus of elasticity and rigidity in different postures, material engineering is particularly important. By controlling the parameters of polymeric synthetic materials, it is possible to find natural or synthetic materials with similar mechanical parameters that will help to meet the required mechanical properties of the disc while stem cells are transplanted. Growth factors play a non-negligible role in the regulation and induction of the regeneration process, and through the study of growth factors, it is possible to regulate the proliferation and differentiation of stem cells according to the desired parameters. Therefore, articles dealing with these topics are the most cited and the hotspot for research on regenerative repair of intervertebral discs.

The most-cited article in IVD repair is the work in 2003 by Sakai and Mochida [14]; this study showed the hypothesize that the maintenance of proteoglycan content in the disc could be achieved by avoiding the depletion of nucleus pulposus and preserving the structure of the annulus, which is a primary factor of decelerating the disc degeneration, and the stem cell level was the best consideration to solve this problem. They took the rabbits with induced discs degeneration as animal model, which autologous MSCs (mesenchymal stem cells) embedded in Atelocollagen((R)) gel were transplanted into the discs. The results demonstrated that the MSCs transplantation is effective in decelerating disc degeneration. The Atelocollagen((R)) gel served as an important carrier of MSCs in transplantation, permitting proliferation, matrix synthesis and differentiation of MSCs. This idea of combining stem cells with cellular scaffold as a strategy for disc repair is widely used and has been shown to be effective. Proteoglycan expression has also become one of the valid indicators for the success of the repair because of the important role it plays in the structure and function of nucleus pulposus and annulus [15].

The second most-cited article is also the oldest in the top 100 list, which is from 1991 by Thompson [16]; this paper describe the stimulation of intervertebral disc by growth factors on mature canine. The study divided the discs into three regions: annular, transitional, and nuclear, and devise a tissue culture system for them. The culture system was perturbed by plasma-derived equine serum, fetal calf serum, insulin-like growth factor-1, epidermal growth factor fibroblast growth factor, and transforming growth factor-beta. They finally found the transforming growth factor-β and epidermal growth factor elicited the greater proliferative response than fibroblast growth factor, more than that, the nucleus and transition zone responded more than anulus to the growth factors. This classic work laid the foundation for growth factors and cell biology in disc repair. More profound and direct evidence of the prominent role of transforming factor-β in stimulating the proliferation and differentiation of disc cells has been provided in numerous studies since then and inspired us to consider adding and loading growth factors to play an important assist role while performing stem cell scaffold to repair discs.

The third most-cited article was the 2006 work of Sakai and Mochida [17]. They transplanted the LacZ expressing MSCs to rabbit IVDs 2 weeks after induction of degeneration. Unlike the study reported in 2003, this experiment set up two control groups, including normal controls (NC) without operations and sham operated with only disc degeneration being induced. Then, the disc height by plain radiograph, T2-weighted signal intensity in MRI, histology, immunohistochemistry and matrix-associated gene expressions were evaluated between them. The results confirmed that the MSC group showed an absolute advantage over the other two groups in terms of preservation of disc structure and accumulation of proteoglycans. Therefore, demonstrated MSCs could serve as a valuable resource in cell transplantation therapy for IVD disease. MSC research in tissue engineering for disc repair is a landmark development in this topic, which has led to the involvement of regenerative medicine in the repair of discs where the shortcomings of conventional sutures and simple resection are compensated by the biological effects of MSC. IN the 100 most-cited articles, 21 were related to MSC. Through observation, there have also been many articles attempting to repair discs through other types of progenitor cells, such as autologous cultured disc-derived chondrocytes (ADCT).

Regenerative medicine and the application of stem cells was the most popular topic in the top 100 articles with a total of 34 works dedicated to it. In the past exploration of stem cell regeneration for the treatment of disc defects, stem cell attempts began with ADCT, some studies have shown it contribute to the repair of discs, but there are significant limitations in its application, due to the utility of such cells was limited by the difficulties with graft procurement, harvest site morbidity, and functionality [18]. There are 7 articles on ADCT. The most important article was the study by Ganey in 2003, demonstrated that the autologous chondrocyte transplantation is technically feasible and biologically relevant to repairing disc damage and retarding disc degeneration. Afterwards, the focus of progenitor cell selection shifts to the application of MSC (mesenchymal stem cells). The number of articles on MSC applications was largest, nearly 22 papers. MSCs contain stem cells and possess the ability to regenerate bone, cartilage, and fibrous tissues. The studies were broadly divided into vitro culture tests and in vivo degenerative model intervention tests. MSCs were loaded into the disc environment in a variety of ways, including direct injection, loading via cellular scaffolds, etc. After a few weeks, the height of the intervertebral gap was assessed by X-ray, disc water content was assessed by T2-weighted term of MRI, the proteoglycan and collagen content was assessed by proteomics. The majority of trials have confirmed that MSC has excellent results in repairing intervertebral disc defects, restoring disc structure and function, and potentially delaying disc degeneration [14]. The most influential between the 22 articles, already mentioned above, was written by Sakai and Mochida. The strategy was MSCs and Atelocollagen((R)) gel to decelerate the disc degeneration. In addition to this, there are many studies exploring the application of other progenitor cells in intervertebral disc repair, with 3 out of 100 articles studied the adipose tissue-derived stem cells. The repair of a damaged disc is possible using autologous adipose tissue derived stem and regenerative cells (ADRCs), and three out of 100 articles addressed the application of notochordal cell (NC) has also been found contributes to the phenotypic differentiation of MSC towards nucleus pulposus (NP) [19].

The second most popular topic out of the 100 articles was Biomaterials and Cellular Scaffolds, which conducted by 31 articles. Biomolecular materials can be used to repair defects in the fibrous ring and medullary nucleus, restore the biomechanical structure and function of the intervertebral disc, and serve as a carrier for loading delivery cells in the cell therapy process, enhancing the effect of progenitor cells regeneration therapy and drug-assisted therapy such as growth factors. Among the 100 articles analyzed, the most used material is hydrogel, which is used by 11 articles. This exhibits the following properties: (1) it is highly plastic and can be used to repair irregular disc defects; (2) it can be synthesized to include sensitive components such as photosensitive components and temperature-sensitive components; and would undergo structural changes through environmental changes, which can help ensure the biomechanical support of the defect site after repair while filling and repairing; these properties make hydrogel one of the most common choices of cellular scaffold materials. Hydrogel plays a different role in the repair of different parts of the intervertebral disc. It has been demonstrated that hydrogels are superior in restoring the structure of the nucleus. In contrast, the role of hydrogels in annulus fibrous repair is still being explored due to the special laminar structure and the higher intensity biomechanical requirements of it. The most-cited article for hydrogel applications was published by Richardson in 2008, which described the trend of human MSC differentiation towards NP cells on chitosan-glycerophosphate hydrogels. In annulus fibrous repair, many attempts have been made in previous studies, such as silk [20], ABS and PLA Scaffold [21], and a cell scaffold made by chitosan salts cross-linked to Genipin [22].

The third most common topic published in the top 100 IVD repair articles was application of growth factors in IVD regenerative therapy. There are 26 articles related to it. The most-cited article is still the classic 1991 article by Thompson, already mentioned above. A significant role of TGF-β in disc regeneration was confirmed in subsequent explorations. In addition, studies have focused on the role of factors such as interleukin-1 (IL-1), osteogenic protein-1 (OP-1), and bone morphogenetic protein-7 (BMP-7).

The most recent article, published in 2017, was written by Dowdell [23]; it is a review about intervertebral disk degeneration and repair, which described the biological therapies as a promising treatment modality for DDD that could impact our future management of low back pain. The 100th article on the list written by Coric et al. [24] is a 12-month prospective cohort of the clinical and radiographic results from a study of cell-based therapy in the treatment of lumbar spondylosis with mechanical LBP.

Among the top-cited articles, 41 papers are published between 2005 and 2009. Unlike the bibliometric analysis of other topics, the most-cited articles are not focused on the early part of the chronological range of distribution, as regeneration and tissue repair of the disc are emerging research directions. It is reasonable to speculate that the reason for the sudden burst of high-quality research in IVD repair between the specific time interval is closely related to the breakthrough in regenerative medicine and tissue engineering research. At the same time, we are forced to consider a phenomenon in the bibliometric analysis, called “obliteration by incorporation,” which suggests that many classic articles will be cited by later HF-cited articles, which may be one reason why the chronological distribution of HF-cited articles presents the current results.

The journal with the most published articles is SPINE, and although disc repair involves multiple subject areas, it can be found that research dedicated to this direction still revolves around the treatment of spinal disorders, indicating a vision in disc repair to help provide solutions for more patients with low back pain.

**Fig. 3:**
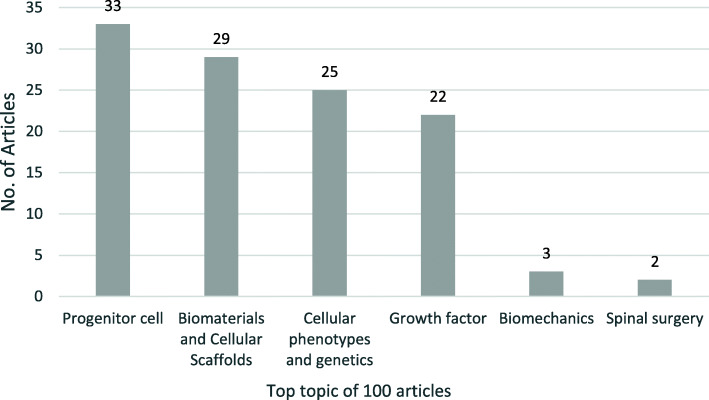
The top topics for the 100 most-cited articles pertaining to IVD repair

To the current authors’ knowledge, this article is the first to identify the 100 articles in IVD repair. This study provides unique insight into the development and trends of this challenging subspecialty within Spine Surgery and Regenerative Medicine in the twentieth and early twenty first centuries. The work identifies topics that were involved most into the ever-growing body of knowledge. Furthermore, we can preliminarily identify research trends in the field of IVD repair at the disciplinary level, and preliminarily identify preferred strategies for IVD repair based on up to three topics involved, as a way of collaborative research in multiple disciplinary areas such as progenitor cell tissue engineering, biomaterials and cellular molecular therapy. The field of stem cells and tissue regeneration is dominated by MSC research, combined with multiple comparative progenitor cell studies. The choice of cellular scaffold material is based on the preparation of hydrogels, and the research of natural materials such as silkworm also foreshadows the current and future research attempts. There is a long history of research on proteins such as growth factors that contribute to IVD repair, and synergistic stem cell therapy will help differentiate the different components of the disc. In addition to this, studies of IVD biomechanics as well as tissue embryology are of particularly importance and will help to select cellular scaffold materials with more complex intervertebral mechanics requirements, and explore the possibility of inducing progenitor cell intervertebral differentiation into annulus fibrous and NP (Fig. 3).

In the process of disc repair, combined with the latest concepts of translational medicine [25], it can be envisaged to work together on a disc repair strategy that meets human needs through interdisciplinary research, and to test the performance of the disc with the help of in vitro tests that simulate the biomechanical environment of the disc in vivo, and to prepare patches according to the operational strategy of clinical disc surgery, analyze the in vivo degradation rate of cellular scaffolds and the differentiation of progenitor cells. The rate of filling and the status of the disc patches were assessed by MRI for follow-up assessment at different postoperative time points. This is fed back to the basic laboratory for parameter adjustment by mechanics, regeneration, and structural recovery in the respective disciplinary laboratories. With regard to possible complications in the post-repair period, timely remediation or revision surgery is performed according to the translational medicine strategy, and it is believed that the regenerative disc repair strategy will be applied earlier in the clinical setting through the translational medicine pathway.

## Conclusions

To the best of our knowledge, this study is the first to identify the top 100 classic articles in IVD repair. This study provides a unique insight into the developments and trends in this challenging new spine surgery subspecialty in the early twentieth and twenty first centuries. This work identifies those individuals, institutions, and countries that have contributed most to the growing body of knowledge, and these past study ideas and disc repair strategies suggest that tissue engineering repair of intervertebral disc is an interdisciplinary research topic involving orthopedics, tissue engineering, biomaterials, such as biochemistry and molecular biology, as well as guiding the future direction of disc repair research. In addition, the 100 articles identified in this study are the most-cited articles, the most influential in the field, and the most memorable. It is reasonable to believe that the regenerative repair of the intervertebral disc will be achieved through the efforts of regenerative medicine, discovering the most appropriate progenitor cells for induction, simulating the microenvironment for the regeneration of intervertebral disc cells with growth factors, combining mechanical simulation with material engineering, preparing cellular scaffolds conforming to the mechanical properties of the fibrous ring and nucleus pulposus using materials such as silk proteins and hydrogels, and exploring in depth the induced changes in physical traits.

## Data Availability

The data collected and analyzed in the article are from WOS, an open access database of scholarly articles, and are properly adopted and collected.

## Abbreviations

IVD: Intervertebral disc
DDD: Degenerative disc disease
WOS: Web of Science
NP: Nucleus pulposus
LBP: Low back pain
HF: High frequency
MSC: Mesenchymal stem cells
ABS: Acrylonitrile butadiene styrene copolymer
PLA: Polylactic acid
NC: Notochordal cell
IL-1: Interleukin-1
OP-1: Osteogenic protein-1 (OP-1)
BMP-7: Bone morphogenetic protein-7
ADRC: Autologous adipose tissue-derived stem and regenerative cells
ADCT: Autologous cultured disc-derived chondrocytes
IF: Impact factor
TGF-β: Transforming growth factor-β
WHO: World Health Organization
